# Are Physicians' USMLE Scores Associated With Patient Mortality? A Multilevel Modeling Approach to USMLE Predictive Validity Evidence

**DOI:** 10.1002/hsr2.71237

**Published:** 2025-09-16

**Authors:** Mohammed A. A. Abulela, Daniel P. Jurich, Alex J. Mechaber, Irina Grabovsky

**Affiliations:** ^1^ Office of Assessment and Evaluation University of Minnesota Medical School Minneapolis Minnesota USA; ^2^ Department of Educational Psychology University of Minnesota Minneapolis Minnesota USA; ^3^ Research Methodology Consulting Center University of Minnesota Minneapolis Minnesota USA; ^4^ National Board of Medical Examiners Philadelphia Pennsylvania USA

**Keywords:** mortality, multilevel modeling, patient outcomes, predictive validity, USMLE

## Abstract

**Background and Aims:**

Validity evidence for high‐stakes licensing examinations, particularly the association between exam scores and clinical practice outcomes, is essential for supporting valid score‐based inferences. However, a gap existed in understanding how variability in patient in‐hospital mortality is partitioned between physicians and hospitals and how physicians' USMLE composite scores are associated with patient mortality using multilevel modeling. Thus, we examined variability in patient in‐hospital mortality between physicians and hospitals and investigated the association between physicians USMLE composite scores and patient mortality, controlling for patient, physician, and hospital characteristics.

**Methods:**

Hospitalization data were sourced from the American Medical Association, USMLE program, and Pennsylvania Health Care Cost Containment Council. Deidentified data included 150,907 patients nested within 1744 physicians in 170 hospitals. Multilevel logistic regression, including a random intercept and six conditional hierarchical models, was used to account for the nested structure and control for covariates. The seven fitted models were compared descriptively and inferentially.

**Results:**

Approximately 5% and 12% of the variability in patient mortality was attributable to physicians and hospitals, respectively. A one standard deviation increase in the USMLE composite score significantly predicted a 6% reduction in patient mortality (odds ratio 0.94; 95% CI = 0.89, 0.99), controlling for patient, physician, and hospital covariates.

**Conclusion:**

Results highlight variability in mortality attributable to physicians and hospitals and underscore the predictive value of USMLE scores. Higher USMLE composite scores were associated with a lower likelihood of patient in‐hospital mortality, providing additional validity evidence for the exam and supporting its use for licensure decisions.

## 1 Introduction

To practice medicine in the US, candidates need to pass a series of licensing examinations in addition to earning their MDs. “The United States Medical Licensing Examination (USMLE) is a three‐step examination for medical licensing in the US. The USMLE assesses a physician's ability to apply knowledge, concepts, and principles, and to demonstrate fundamental patient‐centered skills that are important in health and disease and that constitute the basis of safe and effective patient care” [1]. State medical boards utilize the USMLE scores as the main assessment tool to assist in making licensing decisions. Additionally, others have utilized it for medical students' learning assessment and program evaluation [2].

Because it is a high‐stakes assessment, multiple sources of validity evidence have been rigorously collected to support its score interpretation and uses. Using data from US medical schools and international medical graduates (IMG), prior researchers have established strong validity evidence for the USMLE Step scores based on test content (i.e., rating clinical relevance) and associations with other variables (e.g., passing the American Board of Internal Medicine), among others [3, 4, 5, 6, 7]. Recognizing USMLE as an integrated three‐part examination, Norcini and colleagues used the average converted USMLE score in their research to measure physicians' medical competency in predicting patient mortality. The authors used a large data set of hospitalizations (*N* = 196,881) in the state of PA. They utilized generalized estimating equations and demonstrated that an increase by one standard deviation of the average converted USMLE score was associated with a 5.51% reduction in the odds of patient in‐hospital mortality for several high‐risk medical conditions [8].

We noted, however, that the data used in the Norcini and colleagues' study exhibited a strong clustering (i.e., grouping) structure with patients nested within physicians and physicians nested within hospitals. This type of grouping, often referred to as a hierarchical or multilevel structure, suggested the need for multilevel modeling methods. Such methods allow researchers to estimate how much variability in patients' in‐hospital mortality could be attributed to physicians and hospitals, respectively, which is an important but often overlooked aspect of hospitalization records. Second, it is important to further extend predictive validity inferences for the USMLE, and in particular, to predict patient's in‐hospital mortality while controlling for some physicians', hospitals', and patient characteristics, and to obtain unbiased parameter estimates [9, 10, 11]. Thus, we extend prior research by utilizing multilevel modeling to discern the extent to which variations in mortality outcomes can be attributed to disparities between physicians and hospitals and to add to the body of existing predictive validity evidence for the USMLE. The purpose of the present study was to augment Norcini and colleagues' study by answering the following research questions using multilevel modeling:

1. Does the likelihood of patient mortality vary from one physician to another and from one hospital to another?
2. Are physicians' USMLE composite scores associated with patient in‐hospital mortality after controlling for some patient, physician, and hospital characteristics?

## 2 Methods

### 2.1 Data Sources

To conduct a retrospective cohort study of hospitalizations in Pennsylvania from January 1, 2017 to December 31, 2019, Norcini and colleagues obtained the original data from the three sources: (a) the American Medical Association (AMA), (b) the USMLE program, and (c) the Pennsylvania Health Care Cost Containment Council (PHC4), for hospitalizations in Pennsylvania from January 1, 2017 to December 31, 2019 [8]. We utilized the same data, where the authors detailed how the three data sets were linked using physician and patient unique IDs and other demographic variables. The AMA Physician Masterfile includes primary information for practicing physicians, the USMLE program dataset includes each physician's licensing examination information such as USMLE scores, and the PHC4 includes patient data such as condition, mortality, and length of stay, among others, in addition to the attending physician identifier, resulting in a sizable dataset (*N* = 234,192).

This study was conducted by researchers within the National Board of Medical Examiners (NBME), which securely maintains and governs access to USMLE data. The dataset used was fully deidentified before analysis, and only two authorized members of the research team accessed the data for this study. The project was conducted in accordance with NBME's internal policies for operational research and ethical review. As part of NBME's ongoing validity research program, such studies are conducted to evaluate and support the appropriate interpretation and use of USMLE scores.

The study data included hospitalizations with acute myocardial infarction (AMI), chronic obstructive pulmonary disease (COPD), heart failure (HF), pneumonia, and stroke, dating from January 1, 2017, to December 31, 2019. These five conditions were selected since they are prevalent among patient hospitalizations and are considered indicators of the quality of care [12]. We imposed additional selection criteria on the data to stay consistent with our statistical model and study design. Because some patients (*n* = 26,732) had more than one hospitalization record, we introduced an indicator for the number of hospital visits for every patient. Since the number of hospitalizations can be potentially related to the specific illness causing hospitalization for the patient, we used it as a patient‐level covariate in the multilevel analysis to control for the impact of the varying number of patient hospitalizations. Similarly, because some physicians (*n* = 528) worked in more than one hospital, we created a variable quantifying the number of hospitals where each physician worked and used it as a physician‐level covariate to control for the variable impact of how many locations physicians worked at. This was done to control for the potential confounding effect of varying types of work experience for physicians included in the study.

To allow for the comparison of candidate models with each other, we used the same dataset for all analyses, where we additionally removed records where physicians were missing board certification data and/or sex (*n* = 1566), or patients were missing age and mortality information, or those with the extreme number of hospital visits (e.g., *n* < 3 and *n *> 25). This yielded a final dataset with 150,907 patients nested within 1744 physicians working in 170 hospitals (see Table 1).

**Table 1:** Patient descriptions for each of the studied conditions.

	AMI	HF	Pneumonia	COPD	Stroke	Total
Count: *N* (%)	17,255 (11.4)	44,994 (29.8)	39,809 (26.4)	28,946 (19.2)	19,903 (13.2)	150,907 (100)
Patient age (years): mean (SD)						
Total	69.1 (14.1)	75.4 (13.5)	70.3 (17.3)	69.0 (11.5)	72.5 (13.9)	71.7 (14.6)
Patient sex: *N* (%)						
Female	7115 (41.2)	22,432 (49.9)	20,199 (50.7)	16,498 (57.0)	10,199 (51.2)	76,443 (50.7)
Male	10,140 (58.8)	22,562 (50.1)	19,610 (49.3)	12,448 (43.0)	9704 (48.8)	74,464 (49.3)
Total	17,255 (11.4)	44,994 (29.8)	39,809 (26.4)	28,946 (19.2)	19,903 (13.2)	150,907 (100)
Patient race: *N* (%)						
White, non‐hispanic	14,736 (85.4)	35,888 (79.8)	33,372 (83.8)	23,806 (82.2)	16,273 (81.8)	124,075 (82.2)
Other	2519 (14.6)	9106 (20.2)	6437 (16.2)	5140 (17.8)	3630 (18.2)	26,832 (17.8)
Total	17,255 (11.4)	44,994 (29.8)	39,809 (26.4)	28,946 (19.2)	19,903 (13.2)	150,907 (100)
Patient location: *N* (%)						
Non‐rural	11,211 (65.0)	32,489(72.2)	27,553 (69.2)	20,127 (69.5)	14,421 (72.5)	105,801 (70.1)
Rural	6044 (35.0)	12,505 (27.8)	12,256 (30.8)	8819 (30.5)	5482 (27.5)	45,106 (29.9)
Total	17,255 (11.4)	44,994 (29.8)	39,809 (26.4)	28,946 (19.2)	39,809 (13.2)	150,907 (100)
Physician sex: *N* (%)						
Female	5155 (29.9)	15,178 (33.7)	13,386 (33.6)	9293 (32.1)	6764 (34.0)	49,776 (33.0)
Male	12,100 (70.1)	29,816 (66.3)	19,653 (67.9)	19,653 (67.9)	13,139 (66.0)	101,131 (67.0)
Total	17,255 (11.4)	44,994 (29.8)	39,809 (26.4)	28,946 (19.2)	19,903 (13.2)	150,907 (100)
						
Type of physician's medical school: *N* (%)						
USMG	87,692 (65.6)	87,692 (65.6)	73,332 (66.0)	81,306 (66.7)	86,270 (65.9)	99,938 (66.2)
IMG	45,960 (34.4)	34,761 (32.8)	37,766 (34.0)	40,655 (33.3)	44,734 (34.1)	50,969 (33.8)
Total	17,255 (11.4)	44,994 (29.8)	39,809 (26.4)	28,946 (19.2)	19,903 (13.2)	150,907 (100)
Number of visits: *N* (%)						
	1.1 (0.4)	1.37 (0.9)	1.18 (0.6)	1.47 (1.1)	1.09 (0.4)	1.28 (0.8)
Comorbidity conditions: Mean (SD)						
	1.6 (1.3)	2.8 (1.2)	1.7 (1.3)	1.6 (1.2)	1.9 (1.3)	2.0 (1.3)
Facility volume/1000: Mean (SD)						
	4.6 (2.4)	4.8 (2.6)	4.6 (2.6)	4.4 (2.6)	5.0 (2.7)	4.7 (2.6)
Mortality: *N* (%)						
	551 (3.2)	1152 (2.6)	1316 (3.3)	584 (2.0)	418 (2.1)	4021 (2.7)

Abbreviations: AMI, acute myocardial infarction; COPD, chronic obstructive pulmonary disease; HF, heart failure.

### 2.2 Outcome Variable

In‐hospital mortality was the outcome variable, coded 1 for records with in‐hospital mortality (*n* = 4021) and 0 otherwise. The study sample was constructed to include hospitalization records for all patients with in‐hospital mortality outcomes equal to 1. This way, the record associated with the physician who was the last treating patients with observed hospital mortality was included in the study. At the same time, for patients with no actual mortality observed during their hospital stays, we selected one hospitalization record randomly from all available outcomes with mortality equal to 0. We did so to help with model convergence issues resulting from the patients' wide range of hospitalization records. It should be noted that the retained record for patients with mortality equal to 0 was randomly selected from their set of hospitalizations (to ensure confidentiality within the dataset, no timing information was available for visits, so their last visit could not be retained in the same manner as the patients who experienced mortality on their last visit). Accordingly, the final study sample contained *n* = 4021 or 2.66% of the total, with an in‐hospital mortality measure of 1, and *n* = 146,886, or 97.34% of the sample, with in‐hospital mortality equal to 0.

### 2.3 Independent Variables

We included several independent variables in the model describing patient, physician, and hospital characteristics to investigate whether the association of our focal predictor (i.e., the USMLE standardized composite score) with patient mortality holds after controlling for potential confounders. Patient variables included age, number of hospital visits, condition (e.g., AMI, COPD, HF, Pneumonia, and Stroke, with AMI as the reference group), sex (male as the reference group), race (white, non‐Hispanic as the reference group), the type of the home county (non‐rural as the reference group), and the comorbidity measure, which is an index of patient sickness, ranging from 0 to 9 as severity increases [8].

As for participating physicians, the focal predictor was the standardized USMLE composite score obtained by standardizing the individual Step exam scores (i.e., scaling the scores using the study sample mean and standard deviation) and averaging them. This standardized score was treated as a continuous predictor to assess its association with mortality. Other physician covariates included initial board certification (uncertified as the reference group), sex (female as the reference group), international or domestic status of medical graduates (US medical graduates as the reference group), number of hospitals where a physician worked, and volume (i.e., the number of patients with the five conditions treated by the same physician during the study period). Similarly, the number of admitted patients with the five conditions during the study period in each hospital was calculated to create a continuous measure for each hospital's volume to serve as a hospital‐level covariate.

### 2.4 Data Analysis

Considering the binary nature of the outcome variable and the hierarchical structure of the data (i.e., patients nested with physicians nested within hospitals), we implemented a generalized linear mixed effects modeling (GLMM) framework. Specifically, we applied the multilevel logistic regression using the binomial distribution and the logit link function. We fitted a three‐level random intercepts model (i.e., a “no predictor” or null model) and six conditional models (i.e., models with predictors). The former aimed to answer the first research question via calculating the intraclass correlation coefficient (ICC) while disaggregating the variation in patient mortality between physicians (level‐2) and hospitals (level‐3) and consequently assess the need for multilevel modeling. To address the second research question, six conditional models were fitted to examine the association between the USMLE composite score and patient mortality, while controlling for selected patient, physician, and hospital characteristics. Statistical significance was defined as *p* < 0.05. For detailed information on model building, ICC calculations, and the final model equations, refer to Appendix A.

#### 2.4.1 Centering Covariates and Log‐Odds Interpretation

To obtain stable parameter estimates for the fitted models, improve the interpretation of the intercept, particularly when predictors do not have a meaningful zero value (e.g., age), and reduce collinearity among random intercepts, patient covariates were group‐mean centered, whereas physicians and hospitals were grand‐mean centered, consistent with methodological recommendations [13, 14, 15]. To facilitate the log‐odds interpretation, we reported the odds ratio and 95% confidence interval, which reflect the change in odds of mortality per 1 SD increase in the USMLE score for the final model [16].

#### 2.4.2 Comparing Nested Models

In the present analysis, we compared five pairs of nested models descriptively and inferentially. For the first, we utilized the Akaike information criterion (AIC) and Bayesian information criterion (BIC), with lower values indicating a better fit. Inferentially, we used the likelihood ratio test (LRT), which takes a *χ* ^2^ distribution with degrees of freedom equal to the difference between the estimated numbers of parameters in the two compared models. This was feasible since using maximum likelihood estimation allowed for comparing nested models with different fixed effects [17].

#### 2.4.3 Model Checking

Checking model assumptions has been necessary to validate statistical conclusions [18]. An important component of GLMMs diagnostics is checking for the overdispersion (i.e., the variance is larger than the mean), since prior researchers have found this circumstance to be associated with underestimated standard errors and, consequently, inflated Type I error rates [19].

We conducted all multilevel logistic regression analyses using the *glmmTMB* package in *R* [20, 21]. As part of the sensitivity analysis and cross‐validation of results, we compared results with those obtained from the *lme4* package [22], which were substantially similar. For the variance explained in patient mortality, we reported the marginal *R* ^*2*^ and conditional *R* ^*2*^ obtained using the *sjPlot* package [23], which considers the explained variance attributed to fixed and both the fixed and random effects, respectively [24].

## 3 Results

We presented the study results in the sequence of the research questions investigated in the study and in accordance with the guidelines for reporting multilevel models in general and GLMMs in particular [25, 26].

### 3.1 Partitioning Variability in the Likelihood of Patient Mortality Between Physicians and Hospitals

Results of the unconditional random intercepts model revealed that the ICC estimates were 0.05 and 0.12, indicating that 5% and 12% of the average likelihood of patient mortality can be attributed to physicians and hospitals, respectively. These results illustrated the need to consider multilevel modeling to account for data structure to obtain robust results and consequently draw valid statistical‐based inferences.

### 3.2 Association Between USMLE Composite Score and Patient In‐Hospital Mortality

Before answering the second research question and interpreting results of the final selected model, we presented the comparison criteria of the seven fitted models as shown in Table 2.

**Table 2:** Comparison of fitted models using AIC, BIC, and the LRT.

Comparison criteria	Fitted models						
	Model 1 (unconditional)	Model 2 (step 2 CK)	Model 3 (conditions)	Model 4 (patient)	Model 5 (physician)	Model 6 (hospital)	Model 7 (final)
No. of parameters	3	4	8	14	19	20	15
AIC	36,210	36,205	36,068	34,253	34,234	34,229	34,229
BIC	36,240	36,244	36,148	34,392	34,423	34,427	34,378
Deviance	36,204	36,197	36,052	34,225	34,196	34,189	34,199
Compared model pairs	—	M_2_ vs. M_1_	M_3_ vs. M_2_	M_4_ vs. M_3_	M_5_ vs. M_4_	M_6_ vs. M_5_	M_6_ vs. M_7_
LRT_(df)_		7.36_(1)_**	144.11_(4)_***	1827_(6)_***	28.26_(5)_***	7.50_(1)_**	10 _(5)_

*Note:* The symbols ** and *** indicate statistical significance at *p *< 0.01 and *p *< 0.001, respectively.

Abbreviations: AIC, Akaike information criterion; BIC, Bayesian information criterion; df, degrees of freedom; LRT, likelihood ratio test.

### 3.3 Model Selection

For the nested model comparisons, we first verified that AIC criterion (36,210 vs. 36,205) and the LRT (7.36_(1)_, *p* < 0.01), both favored the model with the USMLE composite score as the only predictor (Model 2) over the null model (Model 1). Thus, results for Model 2, compared to the unconditional model, provided descriptive and inferential evidence that adding the USMLE composite score improved the model‐data fit. Similarly, we verified that in Model 3 (including patient conditions), Model 4 (including patient characteristics), Model 5 (including physicians' characteristics), and Model 6 (including Hospital characteristics), both AIC and the LRT criteria favored the models with more covariates added. However, since BIC penalizes models with more estimated parameters, the patient model (Model 4) fitted the data descriptively better than both the physician (Model 5) and the hospital models (Model 6). Lastly, to arrive at the final model (Model 7), we removed five nonsignificant covariates from Model 6. Model 7 included the physician's USMLE composite score (the focal predictor), patient clinical condition, comorbidity, age, number of visits, race, physician's gender, physician's international status, physician's patient volume, and hospital's volume. Based on the AIC criterion, both models fit the data similarly (34,229 vs. 34,229). Additionally, the final model fitted the data better based on the BIC index (34,378 vs. 34,427). Inferentially, the final parsimonious model fitted the data better than the hospital model, since the *χ* ^2^ was not significant, meaning that removing the nonsignificant predictors from the final model does not improve the fit of the hospital model compared to the final selected model. To ensure statistical conclusion validity, we checked the overdispersion assumption of the final selected model, and it was not violated.

### 3.4 Results of the Final Selected Model

In addition to the unconditional random intercept model results, Table 3 includes results of the six conditional‐fitted multilevel logistic regression models with patient in‐hospital mortality as the outcome variable. In addition to all fitted model regression estimates, we also reported the final model odds ratios and 95% confidence intervals to ease interpretation.

**Table 3:** Multilevel logistic regression models predicting patient mortality using USMLE composite score controlling for patient, physician, and hospital characteristics.

Data levels	Predictors	Fitted models and their estimates							Odds ratio	Odds ratio 95% CI	
		Unconditional	Step 2 CK	Conditions	Patient	Physician	Hospital	Final		LL	UL
	Intercept	−3.71***	−3.71***	−3.73***	−4.40***	−0.42***	−4.54***	−4.54***	0.01	0.01	0.01
	USMLE score		−0.07**	−0.07**	−0.08**	−0.06**	−0.06*	−0.06**	0.94	0.89	0.99
Patient	HF			−0.12*	−0.62**	−0.62***	−0.62***	−0.59***	0.56	0.51	0.60
	COPD			−0.45***	−0.45***	−0.45***	−0.45***	−0.42***	0.66	0.60	0.73
	PNU			0.07	−0.05	−0.05	−0.050	—	—	—	
	Stroke			−0.39***	−0.55***	−0.55***	−0.55***	−0.51***	0.60	0.53	0.67
	CMB				0.21***	0.21***	0.21***	0.21***	1.24	1.21	1.27
	No. visits				0.22***	0.22***	0.22***	0.22***	1.24	1.20	1.28
	Age				0.05***	0.05***	0.05***	0.05***	1.05	1.05	1.05
	Sex				0.059	0.06	0.06	—	—	—	
	Race				0.14*	0.14*	0.14*	0.15*	1.16	1.03	1.30
	Location				0.12*	0.12*	0.12	—	—	—	
Physician	Volume					−0.01**	−0.00**	−0.00**	0.99	0.99	1.00
	Certification					−0.15	−0.14	—	—	—	
	IMG					−0.19***	−0.19***	−0.19***	0.83	0.74	0.92
	Sex					−0.14**	−0.14**	−0.14**	0.87	0.79	0.95
	No. hospitals					−0.00	−0.00	—	—	—	
Hospital	Volume						−0.07**	−0.07**	0.93	0.88	−0.99
Random variance	Physicians	0.19	0.19	0.17	0.21	0.20	0.20	0.20			
	Hospitals	0.48	0.47	0.50	0.53	0.51	0.49	0.48			
*R* ^2^	Marginal	—	0.01	0.01	0.14	0.14	0.15	0.15			
	conditional	0.17	0.17	0.18	0.30	0.30	0.30	0.30			

*Note:* The symbols *, **, and *** indicate statistical significance at *p *< 0.05, *p *< 0.01, and *p *< 0.001, respectively.

Abbreviations: CI, confidence intervals; CK, clinical knowledge; CMB, comorbidity index; COPD, chronic obstructive pulmonary disease; HF, heart failure; IMG, international medical graduate; LL, lower limit; PNU, pneumonia; UL, upper limit; USMLE, United States Medical Licensing Examination.

As noted in Table 3, the physician's USMLE composite score significantly predicted patient mortality, holding the selected patient, physician, and hospital covariates constant. Specifically, a one SD increase in physician USMLE composite score was associated with 6% [(i.e., 1 − 0.94) × 100] reduction in patient mortality (odds ratio = 0.94; 95% CI = 0.89–0.99), holding constant all other patient, physician, and hospital's covariates included in the final model.

Of the patient covariates included, the indicators for HF, COPD, stroke, CMB, number of visits, age, and patient racial group were significant predictors of patient mortality. In more detail, for every one unit increase in CMB and the number of visits, the odds of patient mortality estimate is multiplied by 1.24 (i.e., increases 24%), holding constant all other covariates. Additionally, for every unit increase in patient age and being nonwhite or Hispanic, the odds of patient mortality estimate are multiplied by 1.05 (i.e., increases 5%) and 1.16 times (i.e., increases 16%), with other covariates held constant. The model with patient covariates explained 30% of the variability in patients' mortality.

Turning to the physician and hospital covariates, volume, sex, international status, and hospital volume were statistically significant predictors of patient mortality. However, the conditional *R* ^*2*^ did not increase from that reported in the patient model (*R* ^*2*^ = 0.30), indicating that these covariates had practically negligible effect in predicting mortality, since they explained no additional variability in patient mortality above and beyond what was already explained in the patient model. Overall, considering the effects of all significant covariates, the final model explained approximately 30% of the variability in patient mortality. To summarize, the association between the USMLE composite score and patient in‐hospital mortality was supported, controlling for patient, physician, and hospital characteristics, which in turn adds to the body of validity evidence for the USMLE using multilevel modeling.

## 4 Discussion

As concerns continue to be raised about the extent to which licensing and certification examinations reflect real‐world clinical competence, we investigated how USMLE performance was associated with patient care outcomes, such as in‐hospital mortality. Along with the study's objective to partition variability in the likelihood of patient mortality between physicians and hospitals, we found that 5% and 12% in the variability of the likelihood of patient mortality was attributed to physicians and hospitals, respectively. These results underscore the relatively high variability in patient mortality observed among hospitals in the dataset. Regarding the second objective, using mortality after treatment received by the patients hospitalized with five essential health conditions as an outcome variable, we investigated the association between physicians' USMLE composite score and patient mortality, controlling for some patient, physician, and hospital characteristics. The value of using the USMLE composite score as a focal predictor emphasizes the importance of rigorously assessing “physician's ability to apply knowledge, concepts, and principles, and to demonstrate fundamental patient‐centered skills, that are important in health and disease and that constitute the basis of safe and effective patient care” [1]. All three USMLE Step exams are critical when making physicians' licensing decisions.

To summarize, we found that the attending physicians' higher USMLE composite scores were associated with lower patient mortality rates, controlling for some patient, physician, and hospital characteristics. Specifically, a one SD increase in physicians' USMLE composite scores was associated with an approximately 6% reduction in patient mortality, which agrees with prior research that used the same dataset without exploring the hierarchical structure [8].

### 4.1 Limitations and Future Direction for Research

Despite providing valuable insights to partitioning patient in‐hospital mortality and the USMLE predictive validity evidence, results of the present study have a few limitations. First, our model used one hospitalization record for the patients with several hospitalizations, thus limiting the study dataset. Although a new variable was introduced as a covariate to control for the number of records per patient, it would have been preferred to model all available data. Second, physicians who were ultimately unsuccessful in completing the licensing requirements, including passing all three USMLE Step exams, were not included in the present study, thus restricting the range of scores and likely attenuating our reported association. Stated differently, it should be noted that these results are likely to understate the true association between the USMLE score and physicians' performance in practice, given that physicians with the lowest scores would be unable to be licensed and, therefore, ineligible to practice. Third, we only controlled for available patient, physician, and hospital covariates. Additional studies should examine additional physician and hospital characteristics that were not available to us, such as physician race/ethnicity and years in practice, community versus academic medical center, staff‐to‐patient ratios, availability of specialists, access to updated healthcare technology, and qualifications of nursing staff.

Fourth, it is important to note that the USMLE minimum passing scores have changed over time, and Step 1 transitioned to pass/fail scoring in 2022. As the analyzed dataset includes physicians who likely took the USMLE exams over a broad time range, the numeric scores may reflect differing performance thresholds and normative interpretations. While we utilized standardized composite scores across Steps 1, 2, and 3 to mitigate this issue, caution is warranted in interpreting historical Step scores, particularly in light of recent and ongoing changes to USMLE scoring practices. Future research is needed to evaluate how pass/fail reporting and evolving score thresholds affect the predictive validity of USMLE performance. Last, although our results indicate a statistically significant association between standardized scores and mortality, we did not examine specific score thresholds at which the association becomes significant. This decision was made to preserve the interpretability and statistical power associated with modeling continuous predictors. In future studies, researchers may explore the identification of clinically relevant cutoff scores to support risk stratification or decision‐making in applied contexts.

## 5 Conclusions

The present study has practical implications for the USMLE program and state medical boards. For the former, current results supplement the body of the predictive validity evidence for the USMLE, particularly the extrapolation type of inference [27], which involves the association between the exam performance and that in a larger domain (i.e., quality of patient care). For the latter, stakeholders who utilize USMLE for licensing can feel more confident, based on the additional validity evidence collected in the present study using multilevel modeling, about relying on the measure of physician ability to apply medical knowledge and skills. To conclude, we demonstrated that higher USMLE scores were associated with improved patient mortality for a number of high‐risk medical illnesses treated in a hospital context, which lends more credence to the interpretation and use of the USMLE composite score.

## Author Contributions

**Mohammed A. A. Abulela:** conceptualization, investigation, writing – original draft, methodology, validation, writing – review and editing, software, formal analysis, project administration, data curation, supervision. **Daniel P. Jurich:** writing – review and editing. **Alex J. Mechaber:** writing – review and editing. **Irina Grabovsky:** conceptualization, investigation, writing – review and editing, methodology, validation, project administration, formal analysis, data curation, supervision.

## Ethics Statement

This study falls under the NBME institutional research plan as approved by the American Institutes for Research Review Board. As listed in the IRB documentation, the project is exempt from further IRB review because it does not constitute research or because it does not involve human subjects. The ethical approval reference number is IRB00000436.

## Conflicts of Interest

The authors declare no conflicts of interest.

## Transparency Statement

The lead author as well as all co‐authors affirm that this manuscript is an honest, accurate, and transparent account of the study being reported; that no important aspects of the study have been omitted; and that any discrepancies from the study as planned (and, if relevant, registered) have been explained.

## Data Availability

This is a health sciences project, and data are not available.

## Appendix A The intraclass correlation, model building, and the final model equation

The intraclass correlation coefficient (ICC) can be computed from Equation 1 (Level 2) and 2 (Level 3).

(1)
$$\rho_{2}=\;\frac{\tau_{00}^{\left(2\right)}}{\tau_{00}^{\left(2\right)}+\;\tau_{00}^{\left(3\right)}+\;\sigma^{2}}$$

(2)
$$\rho_{3}=\;\frac{\tau_{00}^{\left(3\right)}}{\tau_{00}^{\left(2\right)}+\;\tau_{00}^{\left(3\right)}+\;\sigma^{2}}$$

where $\rho_{2}$ and $\rho_{3}$ are the ICC quantifying the variability in patient mortality between physicians and hospitals, respectively. On the other side of the equations, $\sigma^{2}$, $\tau_{00}^{\left(2\right)}$, and $\tau_{00}^{\left(3\right)}$ are the variance components associated with patients (Level‐1), physicians, and hospitals, respectively. Since the variance scale factor in logistic regression is scaled to 1, it is worth noting that $\sigma^{2}$ is fixed to $\frac{\pi^{2}}{3}$ or approximately 3.29 [9].

To answer the second research questions, we fitted six conditional models as described in more detail below.

1. The USMLE composite score: predicting patient mortality by the focal predictor (USMLE composite score) only.
2. Patient conditions: predicting patient mortality by the USMLE composite score + HF + COPD + pneumonia + stroke. This model was fitted to investigate if the association between the USMLE composite score and mortality holds, controlling for the five conditions (note that AMI was set as the reference group as stated earlier).
3. Patient characteristics: predicting patient mortality by the USMLE composite score + HF + COPD + pneumonia + stroke + age + comorbidity + number of visits + sex + race + location. This model was fitted to test if the association between the USMLE composite score and mortality holds, controlling for patient conditions and six other patient‐related covariates.
4. Physician characteristics: predicting patient mortality by the USMLE composite score + HF + COPD + pneumonia + stroke + age + comorbidity + number of visits + sex + race + location + certification + physician volume + IMG + number of hospitals + physician sex. This model was fitted to test if the association between the USMLE composite score and mortality holds, controlling for patient conditions and some patient‐ and physician‐related covariates.
5. Hospital characteristics: predicting patient mortality by the USMLE composite score + HF + COPD + pneumonia + stroke + age + comorbidity + number of visits + sex + race + location + certification + physician volume + IMG + number of hospitals + physician sex + hospital volume. This model was fitted to investigate if the association between the USMLE composite score and mortality holds while controlling for patient conditions and some patient, physician, and hospital covariates.
6. The final model: predicting patient mortality by the USMLE composite score + HF + COPD + stroke + age + comorbidity + number of visits + race + physician volume + IMG + physician sex + hospital volume. This model was fitted to check if the association between the USMLE composite score and mortality holds after removing all nonsignificant covariates from model 5 for the sake of parsimony (i.e., simplicity of the final model). The final model is represented in the set of equations below disaggregated by each of the three data levels.

Level‐1 model

(1)
$${ƞ}_{\mathrm{ijk}}=\pi_{0\mathrm{jk}}+\pi_{1\mathrm{jk}}{\mathrm{COPD}}_{\mathrm{ijk}}+\pi_{2\mathrm{jk}}{\mathrm{HF}}_{\mathrm{ijk}}+\pi_{3\mathrm{jk}}{\mathrm{Stroke}}_{\mathrm{ijk}}+\pi_{4\mathrm{jk}}{\mathrm{CMD}}_{\mathrm{ijk}}+\pi_{5\mathrm{jk}}{\mathrm{Visits}}_{\mathrm{ijk}}+\pi_{6\mathrm{jk}}{\mathrm{Age}}_{\mathrm{ijk}}+\pi_{7\mathrm{jk}}{\mathrm{Race}}_{\mathrm{ijk}}+e_{\mathrm{ijk}}$$

Level‐2 model

(2)
$$\pi_{0\mathrm{jk}}=\beta_{00k}+\beta_{01k}\;{\mathrm{USMLE}}_{\mathrm{jk}}+\beta_{02k}\;{\mathrm{Vol}}_{\mathrm{jk}}+\beta_{03k}\;{\mathrm{Male}}_{\mathrm{jk}}+\beta_{04k}\;{\mathrm{IMG}}_{\mathrm{jk}}+r_{0\mathrm{jk}}\pi_{1\mathrm{jk}}=\beta_{10k}\pi_{2\mathrm{jk}}=\beta_{20k}\pi_{3\mathrm{jk}}=\beta_{30k}\pi_{4\mathrm{jk}}=\beta_{40k}\pi_{5\mathrm{jk}}=\beta_{50k}\pi_{6\mathrm{jk}}=\beta_{60k}\pi_{7\mathrm{jk}}=\beta_{70k}$$

Level‐3 model

(3)
$$\beta_{00k}=\gamma_{000}+\gamma_{001}{\mathrm{Vol}}_{k}+u_{00k}\beta_{01k}=\gamma_{010}\beta_{02k}=\gamma_{020}\beta_{03k}=\gamma_{030}\beta_{03k}=\gamma_{040}$$

where ${ƞ}_{\mathrm{ijk}}$ is the log‐odds of the probability of mortality of patient *i* nested within physician *j* nested within hospital *k*, $\pi_{0\mathrm{jk}}$ is the intercept or average log‐odds of mortality for patients treated by physician *j* admitted to hospital *k*, $\beta_{00k}$ is the intercept or average log‐odds of patient mortality in hospital *k*, $\gamma_{000}$ is the overall intercept or average log‐odds of patient mortality across all hospitals. Further, in Level‐2 Model, $\beta_{10k}$, $\beta_{20k}$, $\beta_{30k}$, $\beta_{40k}$, $\beta_{50k}$, $\beta_{60k}$, and $\beta_{70k}$ are, respectively, the average regression slopes associated with patient COPD, HF, stroke, CMD index, number of visits, age, and race. In the Level‐3 Model, $\gamma_{010}$, $\gamma_{020}$, $\gamma_{030}$, and $\gamma_{040}$ are the average regression slopes capturing the impact of physician USMLE composite scores, volume, sex, and IMG status on patient mortality intercepts, respectively, and $\gamma_{001}$is the regression slope capturing the impact of hospital volume on patient mortality at the hospital level. Lastly, $e_{\mathrm{ijk}}$, $r_{0\mathrm{jk}}$, and $u_{00k}$ are the error terms associated with patient *i*, physician *j*, and hospital *k*, respectively.
